# Tumor Budding Unveiled: Clinicopathological Correlations and Survival Insights From a Tertiary Care Cancer Center of India

**DOI:** 10.1155/ijbc/6846485

**Published:** 2026-08-24

**Authors:** Ipsita Dhal, Himani Rai, Neha Singh, Ahmad Raza, Divya Khichi, Isha Makker, Zachariah Chowdhury, Shashikant Patne, Parul tripathi, Ankita Pal, Anuj Gupta, Mayank Tripathi, Bal Krishna Mishra

**Affiliations:** ^1^ Department of Oncopathology, Mahamana Pandit Madan Mohan Malaviya Cancer Centre, Homi Bhabha National Institute, Varanasi, India, hbni.ac.in; ^2^ Department of Pathology, Institute of Medical Sciences, Banaras Hindu University, Varanasi, India, bhu.ac.in; ^3^ Department of Pathology, Artemis Hospital, Gurgaon, New Delhi, India, artemishospitals.com; ^4^ Department of Clinical Research Secretariat, Mahamana Pandit Madan Mohan Malaviya Cancer Centre, Homi Bhabha National Institute, Varanasi, India, hbni.ac.in; ^5^ Department of Medical Oncology, Mahamana Pandit Madan Mohan Malaviya Cancer Centre, Homi Bhabha National Institute, Varanasi, India, hbni.ac.in; ^6^ Department of Surgical Oncology, Mahamana Pandit Madan Mohan Malaviya Cancer Centre, Homi Bhabha National Institute, Varanasi, India, hbni.ac.in

## Abstract

**Background:**

Tumor budding (TB), defined as isolated single cells or small clusters at the invasive front, has emerged as a potential adverse prognostic marker in breast cancer (BC), though its clinical utility remains uncertain.

**Methods:**

This retrospective study included 229 cases of invasive breast carcinoma. TB was assessed on hematoxylin and eosin (H&E) sections, with AE1/AE3 immunostaining in equivocal cases. TB was analyzed both as a continuous and categorical variable for its association with clinicopathological parameters, including lymph node metastasis, lymphovascular invasion (LVI), distant metastasis, tumor size, nodal stage, age, and hormone receptor status. Patients were followed for 5.75 years to evaluate progression‐free survival (PFS) and overall survival (OS).

**Results:**

High tumor budding (HTB) was identified in a significant proportion of cases and showed strong correlation with adverse clinicopathological features, including lymph node positivity, LVI, higher nodal stage, and distant metastasis. HTB was more frequent in hormone receptor–positive tumors and less common in triple‐negative BC. No significant association was observed between TB and PFS or OS during follow‐up.

**Conclusion:**

TB is a cost‐effective histopathological marker associated with adverse clinicopathological features in BC. Although no significant association with survival outcomes was observed in the present cohort, its correlation with established adverse pathological parameters highlights its potential clinical relevance. Larger prospective studies with standardized assessment criteria are needed to further clarify its prognostic significance and clinical utility.

## 1. Introduction

Breast cancer (BC) constitutes a major public health burden globally, being the most prevalent malignancy among women and the second foremost cause of cancer‐related mortality all over the world [1, 2]. Although significant progress has been made in the early detection and treatment of BC, it continues to pose a major public health challenge, largely due to its widespread occurrence and the considerable differences in outcomes among patients [3]. Survival outcomes among patients with BC can differ substantially depending on various factors, including tumor biology, treatment approaches, and individual patient characteristics. Therefore, the identification of reliable prognostic markers and risk factors, which are linked to poorer survival, is essential to enhance clinical decision‐making and optimize patient care [4]. Tumor budding (TB) has emerged as a prognostic marker in various cancers including pancreatic cancer, colorectal cancer, and gastric cancer [5–7]. This is believed to be mediated by epithelial–mesenchymal transition (EMT), a key cellular process in which epithelial cells undergo phenotypic changes to acquire mesenchymal features, promoting enhanced motility and invasiveness [4, 8, 9]. The loss of the epithelial marker E‐cadherin disrupts cell‐to‐cell adhesion and polarity, whereas increased expression of the mesenchymal marker vimentin promotes cytoskeletal reorganization and enhances cell motility. Tumor buds can invade lymphovascular structures, facilitating systemic dissemination to distant sites. With further molecular characterization, TB may emerge as a potential therapeutic target in various cancers [10]. Several studies have established a correlation between TB and aggressive features of BC, including higher histological grade, presence of lymphovascular invasion (LVI), and decreased hormone receptor (HR) expression.

The present study is aimed at studying the TB in the invasive breast carcinoma (IBC) cases and its correlation with clinical and various histopathological parameters and also its impact on survival of these patients.

## 2. Material and Methods

This retrospective study was conducted in the Department of Oncopathology, of a tertiary care cancer center, over a period of 2 years from August 2018 to August 2020 after approval from our Institutional Ethics Committee (IEC No. 11000056). All the cases diagnosed as IBC on resection were retrieved from the archives. All the cases who underwent upfront breast surgery (mastectomy or breast conservation surgery) without receiving any presurgical chemotherapy or radiotherapy were included, whereas the cases in which only core biopsy sample was available or those who received presurgical chemotherapy and/or radiotherapy were excluded from the study. Detailed clinical history, results of relevant investigations including histopathology, immunohistochemistry (IHC) , and radiological data were retrieved and studied. The histopathological slides, along with the relevant IHC slides, from all cases were retrieved for review. All findings, including histomorphology, tumor staging, and the associated immunohistochemical analyses were thoroughly documented.

### 2.1. Evaluation of Tumor Buds

TB is defined as single cells or small clusters of fewer than five cells situated at the invasive front of a tumor [4, 5, 8, 9]. TB was evaluated only at the invasive front of the tumor. Invasive front of the breast carcinoma was identified in the scanner (4x objective). Tumor buds were searched in low power (10x objective). Areas with the highest concentration of tumor buds (hot spot) were selected for scoring, avoiding the areas with extensive necrosis or mucin, if present. The number of tumor buds was counted in the high power field (HPF) (40x objective) and the total number of tumor buds in 10 HPF was documented. We assessed TB independently by both a resident pathologist and a consultant pathologist. In cases with discrepant counts, the slides were reviewed jointly and a consensus tumor bud count was assigned.

### 2.2. Scoring of Tumor Buds [8, 9]

TB was categorized using a cutoff adopted from previously published studies to facilitate interstudy comparability. The tumor buds were graded into two groups: low tumor budding (LTB) group–tumor buds ≤ 20/10 HPF and high tumor budding (HTB) group–tumor buds > 20/10 HPF.

**Figure 1:**
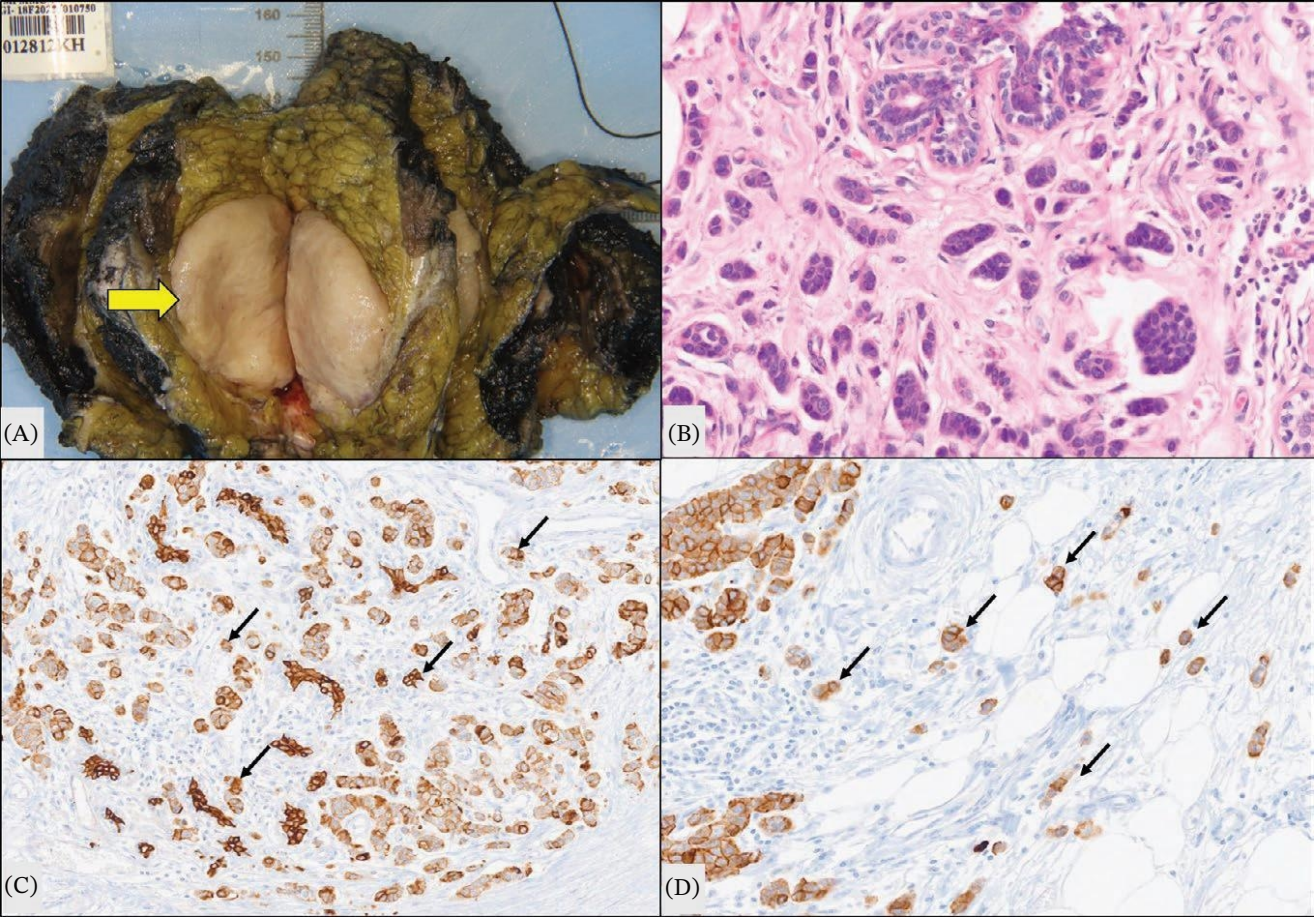
Assessment of tumor budding in invasive breast carcinoma. (A) Gross photograph of a mastectomy specimen showing a grey‐white tumor on cut section (yellow arrow). (B) Tumor budding at the invasive front, characterized by isolated single tumor cells and small clusters comprising fewer than five cells (H&E, ×40). (C) High tumor budding highlighted by pan‐cytokeratin (Pan‐CK) immunostaining (×40). (D) Low tumor budding; tumor buds are indicated by black arrows (H&E, ×40).

The correlation between the number of tumor buds and various clinical and histopathological parameters, including tumor size, grade, LVI, perineural invasion (PNI), necrosis, Ductal carcinoma in situ (DCIS), lymph node (LN) involvement, HR status, as well as clinical factors such as age and tumor size were assessed. Molecular subtypes were assigned using surrogate immunohistochemical classification based on ER, PR, and HER2 status. Tumors were classified as hormone receptor‐positive/HER2‐negative (HR+/HER2−), hormone receptor‐positive/HER2‐positive (HR+/HER2+), HER2‐enriched (ER−/PR−/HER2+), or triple‐negative BC (triple‐negative breast cancer [TNBC]; ER−/PR−/HER2−). The collected data were organized into tables and analyzed using appropriate statistical methods to evaluate data distribution. Representative examples of LTB and HTB identified on H&E sections and highlighted by pan‐cytokeratin immunostaining are shown in Figure 1.

### 2.3. Statistical Analysis

The patients′ baseline characteristics and clinical features were represented using descriptive statistics. Categorical variables were presented using frequencies and percentages. Continuous variables were presented using median and range, as the data showed nonnormal distribution when tested using the Kolmogorov–Smirnov test. Associations between categorical clinicopathological parameters and TB grade (LTB and HTB) were evaluated using the chi‐square test or Fisher′s exact test, as appropriate. In addition, TB was analyzed as a continuous variable. Continuous associations between TB counts and clinicopathological parameters were assessed using Spearman rank correlation analysis, and differences in TB counts between groups were evaluated using the Mann–Whitney *U* test. The progression‐free survival (PFS) and overall survival (OS) were estimated using the Kaplan–Meier method with a 95% confidence interval (CI). Prognostic factors influencing PFS and OS were assessed using a univariable and multivariable Cox proportional hazard model, represented using hazard ratio with a 95% CI. A clinically adjusted multivariable Cox proportional hazards model was constructed by including TB as a continuous variable together with established prognostic covariates, namely age, pathological T stage (pT stage), pathological nodal stage (pN stage), histological grade, LVI, and molecular subtype (HR+/HER2−, HR+/HER2+, HER2‐enriched, and TNBC), irrespective of their univariable statistical significance. All the covariates included in the Cox model were checked for Cox pH assumptions using Schoenfeld residuals. Both PFS and OS were compared between the TB groups using the log‐rank test. The PFS was defined as the time to event end point from the date of registration till the date of progression or death due to any cause, and OS was defined as the time to event end point from the date of registration till the date of death due to any cause. All the statistical tests were two‐sided, and *p* < 0.05 was considered statistically significant. Statistical analyses were performed using SPSS 30.0 software (IBM SPSS Software, Armonk, New York) or R software (Version 4.5.1).

## 3. Results

This retrospective study included 229 patients with IBC over a period of 2 years. Of these, 224 (97.8%) were females and five were males. The age ranged from 25 to 80 years, with a median age of 50 years.

Baseline demographic and clinicopathological characteristics of the study cohort are summarized in Table 1. Invasive breast carcinoma‐no special type (IBC‐NST) was the predominant histological subtype. Most tumors were Nottingham Grade 3. Associated DCIS, predominantly high grade, was identified in a substantial proportion of cases. LVI, PNI, and necrosis were observed in a subset of tumors. Most tumors were staged as pT2, whereas nodal status was predominantly N0. HR positivity for ER and PR was observed in more than half of the cases, whereas HER2/Neu positivity was identified in approximately one‐fourth of cases. TNBC constituted nearly one‐fourth of the cohort. Molecular subtyping based on ER, PR, and HER2 status demonstrated that the HR+/HER2− subtype was the most common, comprising 120 cases (52.4%), followed by TNBC in 57 cases (24.9%), HER2‐enriched subtype in 28 cases (12.2%), and HR+/HER2+ subtype in 24 cases (10.5%).

**Table 1:** Baseline demographics and clinical characteristics of the study cohort (*N* = 229).

Parameter	Total cohort (*N* = 229)
Age (years), *median* (*range*)	50 (25–80)
Age group, *n* (%)	
≤ 50 years	119 (52%)
> 50 years	110 (48%)
Maximum tumor size (cm), *median* (*range*)	4 (0.10–16)
Tumor size category, *n* (%)	
< 2 cm	18 (8%)
2–5 cm	174 (76%)
> 5 cm	37 (16%)
Tumor type, *n* (%)	
Invasive ductal carcinoma (IDC)	212 (93%)
Invasive lobular carcinoma (ILC)	5 (2%)
Metaplastic carcinoma	5 (2%)
Mucinous carcinoma	3 (1%)
Invasive papillary	2 (1%)
Solid papillary	1 (0.4%)
Tubular carcinoma	1 (0.4%)
Grade of tumor, *n* (%)	
Grade 1	7 (3%)
Grade 2	67 (31%)
Grade 3	144 (66%)
T stage, *n* (%)	
T1	32 (14%)
T2	150 (66%)
T3	31 (14%)
T4	16 (7%)
N stage, *n* (%)	
N0	116 (51%)
N1	58 (25%)
N2	23 (10%)
N3	31 (14%)
NX	1 (0.4%)
Lymph node metastasis, *n* (%)	
Absent	119 (52%)
Present	110 (48%)
Distant metastasis, *n* (%)	
Absent	196 (86%)
Present	33 (14%)
Lymphovascular invasion (LVI), *n* (%)	
Absent	150 (66%)
Present	79 (35%)
Perineural invasion (PNI), *n* (%)	
Absent	201 (88%)
Present	28 (12%)
Necrosis, *n* (%)	
Absent	86 (38%)
Present	143 (62%)
DCIS grade, *n* (%)	
Absent	97 (42%)
Low	1 (0.4%)
Intermediate	38 (17%)
High	93 (41%)
Estrogen receptor (ER), *n* (%)	
Negative	97 (42%)
Positive	132 (58%)
Progesterone receptor (PR), *n* (%)	
Negative	102 (45%)
Positive	127 (56%)
HER2/Neu, *n* (%)	
Negative	171 (75%)
Positive	58 (25%)
Triple‐negative breast cancer (TNBC), *n* (%)	
No	172 (75%)
Yes	57 (25%)
Molecular subtype	
HR+/HER2−	120 (52.4%)
HR+/HER2+	24 (10.5%)
HER2‐enriched	28 (12.2%)
TNBC	57 (24.9%)
Tumor budding grade, *n* (%)	
Low	107 (47%)
High	122 (53%)

HTB was noted in 122 cases (53%), whereas 107 cases (47%) demonstrated LTB. TB was analyzed in relation to multiple clinicopathological parameters, including patient age, tumor size, LN involvement, LVI, PNI, histological grade, necrosis, HR status, TNM stage, and tumor subtype (Table 2).

**Table 2:** Comparison of patient characteristics with tumor budding grade.

Parameters		Tumor budding *n* (%)		*p* ^∗^	*r*	*p* ^#^
		Low (*n* = 107)	High (*n* = 122)			
Age	*≤ 50* year	57 (53.3%)	62 (50.8%)	0.406	0.04 (−0.09–0.17)	0.55
	*> 50 year*	50 (46.7%)	60 (49.2%)			
Tumor size	*< 2* cm	9 (8.4%)	9 (7.4%)	0.894	0.04 (−0.09–0.17)	0.53
	*2–5* cm	82 (76.6%)	92 (75.4%)			
	*> 5 cm*	16 (15%)	21 (17.2%)			
LN mets	*Present*	43 (40.2%)	67 (54.9%)	**0.024**	0.16 (0.03–0.29)	**0.02**
	*Absent*	64 (59.8%)	55 (45.1%)			
Lymphovascular invasion	*Present*	25 (23.4%)	54 (44.3%)	**< 0.001**	0.20 (0.07–0.33)	**0.002**
	*Absent*	82 (76.6%)	68 (55.7%)			
Perineural invasion	*Present*	9 (8.4%)	19 (15.6%)	0.073	0.14 (0.01–0.27)	**0.03**
	*Absent*	98 (91.6%)	103 (84.4%)			
Grade of tumor	*Grade 1*	4 (4.1%)	3 (2.5%)	0.107^£^	−0.09 (−0.22–0.05)	0.20
	*Grade 2*	23 (23.5%)	44 (36.7%)			
	*Grade 3*	71 (72.4%)	73 (60.8%)			
	*Unknown*	2 (1.9%)	9 (7.4%)			
DCIS grade	*High*	46 (43%)	47 (38.5%)	0.653^£^	−0.05 (−0.18–0.08)	0.60
	*Low*	1 (0.9%)	0 (0%)			
	*Intermediate*	18 (16.8%)	20 (16.4%)			
	*Absent*	42 (39.3%)	55 (45.1%)			
ER	*Positive*	54 (50.5%)	78 (63.9%)	**0.027**	0.17 (0.04–0.30)	**0.01**
	*Negative*	53 (49.5%)	44 (36.1%)			
PR	*Positive*	51 (47.7%)	76 (62.3%)	**0.018**	0.15 (0.02–0.28)	**0.02**
	*Negative*	56 (52.3%)	46 (37.7%)			
Her–2 Neu	*Positive*	26 (24.3%)	32 (26.2%)	0.428	0.05 (−0.09–0.18)	0.47
	*Negative*	81 (75.7%)	90 (73.8%)			
Triple negative breast cancer	*Yes*	33 (30.8%)	24 (19.7%)	**0.036**	−0.14 (−0.27–[−0.007])	**0.03**
	*No*	74 (69.2%)	98 (80.3%)			
Molecular subtype	*HR+/HER2−*	51 (47.7%)	69 (56.6%)	0.237	−0.13 (−0.26–0.006)	0.05
	*HR+/HER2+*	9 (8.4%)	15 (12.3%)			
	*HER2−enriched*	15 (14%)	13 (10.7%)			
	*TNBC*	32 (29.9%)	25 (20.5%)			
Necrosis	*Positive*	65 (60.7%)	78 (63.9%)	0.359	0.003 (−0.13–0.14)	0.96
	*Negative*	42 (39.3%)	44 (36.1%)			
PT stage	*pT1*	18 (16.8%)	14 (11.5%)	0.730	0.07 (−0.06–0.20)	0.28
	*pT2*	68 (63.6%)	82 (67.2%)			
	*pT3*	14 (13.1%)	17 (13.9%)			
	*pT4*	7 (6.5%)	9 (7.4%)			
PN stage	*pN0*	64 (59.8%)	53 (43.4%)	**0.041**	0.20 (0.07–0.33)	**0.002**
	*pN1*	23 (21.5%)	35 (28.7%)			
	*pN2*	11 (10.3%)	12 (9.8%)			
	*pN3*	9 (8.4%)	22 (18%)			
Type of tumor	*IDC*	99 (92.5%)	113 (92.6%)	0.107^£^	0.03 (−0.10–0.17)	0.61
	*ILC*	0 (0%)	5 (4.1%)			
	*Metaplastic*	4 (3.7%)	1 (0.8%)			
	*Mucinous*	2 (1.9%)	1 (0.8%)			
	*Tubular*	1 (0.9%)	0 (0%)			
	*Invasive papillary*	1 (0.9%)	1 (0.8%)			
	*Solid papillary*	0 (0%)	1 (0.8%)			

*Note: r* denotes Spearman‐rank correlation coefficient. Bold *p* values are statistically significant.

^∗^ *p* values were calculated using Fisher Exact test^£^ or chi‐square test.

^#^ *p* values were calculated using Mann–Whitney *U* test.

HTB demonstrated a significant association with LN metastasis (54.9% vs. 40.2% in LTB; *p* = 0.024), LVI (44.3% vs. 23.4%; *p* < 0.001), and pN stage (*p* = 0.041). Continuous analysis of TB showed significant positive correlations with LN metastasis (*r* = 0.16, *p* = 0.02), LVI (*r* = 0.20, *p* = 0.002), PNI (*r* = 0.14, *p* = 0.03), ER positivity (*r* = 0.17, *p* = 0.01), PR positivity (*r* = 0.15, *p* = 0.02), and pN stage (*r* = 0.20, *p* = 0.002). A significant negative correlation was observed between TB and TNBC (*r* = −0.14, *p* = 0.03), indicating that TNBC was relatively less common among tumors with high budding. No significant correlations were identified between TB and age, tumor size, tumor grade, DCIS grade, HER2/Neu status, necrosis, pT stage, or tumor type.

Although the categorical comparison of molecular subtype did not reach statistical significance (*p* = 0.237), HR+/HER2− tumors were more frequent in the HTB group (56.6%) than in the LTB group (47.7%), whereas TNBC was more common in the LTB group (29.9%) than in the HTB group (20.5%).

## 4. Survival Analysis

**Figure 2:**
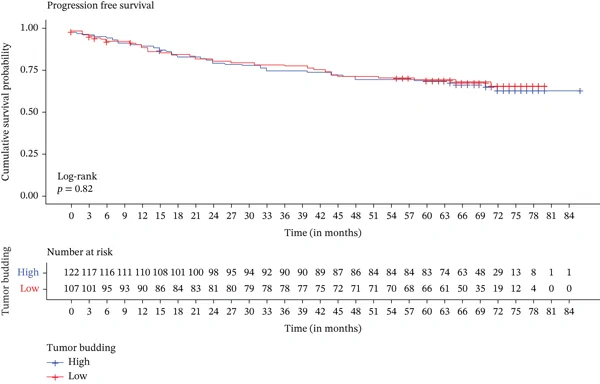
Kaplan–Meier graph depicting outcomes and comparison using log‐rank test for progression‐free survival as endpoints.

**Figure 3:**
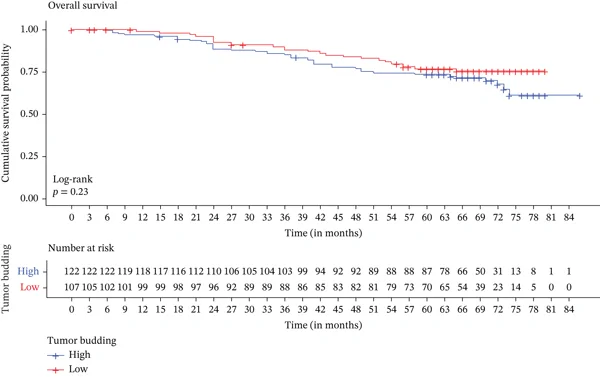
Kaplan–Meier graph depicting outcomes and comparison using log‐rank test for overall survival as endpoints.

The median follow‐up duration of the cohort was 69 months (IQR: 65–73 months), that is, 5.75 years, with a minimum follow‐up of 0 months and a maximum of 86 months. Patients with HTB had a median follow‐up of 70 months (IQR: 66–73 months), whereas patients with LTB had a median follow‐up of 68 months (IQR: 64–72 months). The mean PFS and OS for patients with HTB were 65 months (95% CI: 59–70 months) and 69 months (95% CI: 65–74 months), respectively. The 5‐year PFS and OS rates were 66.7*%* ± 4.3*%* and 71.3*%* ± 4.2*%*, respectively. The mean PFS and OS for patients with LTB were 62 months (95% CI: 56–67 months) and 70 months (95% CI: 66–73 months), respectively. The 5‐year PFS and OS rates were 68*%* ± 4.7*%* and 75.3*%* ± 4.4*%*, respectively. Comparison between HTB and LTB groups showed no statistically significant difference in PFS or OS (*p* = 0.822 and 0.232, respectively). Kaplan–Meier survival curves and comparison using log‐rank test for PFS and OS are shown in Figures 2 and 3.

Multiple clinicopathological factors, including age, pT stage, pN stage, histological grade, LVI, molecular subtype, and TB, were evaluated for their association with PFS and OS using Cox regression analysis. All covariates included in the Cox models satisfied the proportional hazards assumption based on Schoenfeld residual analysis (*p* > 0.05). In addition, TB was analyzed as a continuous variable in the clinically adjusted multivariable Cox model, which included age, pT stage, pN stage, histological grade, LVI, PNI, and molecular subtype irrespective of their univariable statistical significance.

On multivariable Cox regression analysis using a clinically adjusted model, age, TB, pT stage, tumor grade, LVI, and molecular subtype did not demonstrate independent associations with either PFS or OS. Advanced nodal stage (pN3) was the most important independent prognostic factor, showing a significantly higher risk of progression (HR = 2.13, 95% CI: 1.1–4.1, *p* = 0.03) and death (HR = 2.30, 95% CI: 1.1–4.7, *p* = 0.02) compared with pN0 disease. TB, analyzed as a continuous variable, was not independently associated with PFS (HR = 1.00, 95% CI: 0.99–1.00, *p* = 0.99) or OS (HR = 1.00, 95% CI: 0.99–1.00, *p* = 0.81), indicating that TB did not provide independent prognostic information beyond established clinicopathological covariates in the present cohort (Table 3).

**Table 3:** Multivariate Cox proportional hazard regression analysis to assess the factors influencing survival with progression‐free survival and overall survival as endpoints.

Features		PFS		OS	
		HR (95% CI)	*p*	HR (95% CI)	*p*
Age	*≤ 50* year	1		1	
	*> 50 year*	0.80 (0.5–1.3)	0.39	0.99 (0.5–1.8)	0.97
Tumor budding		1 (0.9–1)	0.99	1 (0.99–1)	0.81
pT Stage	*pT1*	1		1	
	*pT2*	0.99 (0.5–2.2)	0.99	0.87 (0.4–2)	0.75
	*pT3*	1.91 (0.8–4.5)	0.14	1.93 (0.8–4.9)	0.17
	*pT4*	1.64 (0.6–4.6)	0.35	2.37 (0.8–6.9)	0.12
pN stage	*pN0*	1		1	
	*pN1*	1.20 (0.6–2.3)	0.58	1.13 (0.5–2.3)	0.74
	*pN2*	0.92 (0.4–2.3)	0.86	0.52 (0.1–1.8)	0.30
	*pN3*	2.13 (1.1–4.1)	0.03	2.30 (1.1–4.7)	0.02
Tumor grade	*Grade 3*	1		1	
	*Grade 1*	0	0.99	0	0.99
	*Grade 2*	1.02 (0.6–1.7)	0.93	0.87 (0.5–1.6)	0.65
LVI	*Absent*	1		1	
	*Present*	0.80 (0.5–1.4)	0.41	0.78 (0.4–1.4)	0.43
Molecular subtype	*HR+/HER2−*	1		1	
	*HR+/HER2+*	1.32 (0.6–2.8)	0.48	1.48 (0.6–3.4)	0.35
	*HER2-enriched*	1.32 (0.6–2.8)	0.47	1.21 (0.5–2.8)	0.65
	*TNBC*	1.26 (0.7–2.4)	0.46	1.14 (0.6–2.3)	0.72

*Note:* All covariates did not violate Cox PH assumptions.

## 5. Discussion

TB has emerged as a potential adverse prognostic marker in various malignancies, particularly in colorectal cancer, where it has been extensively studied [11]. Several studies have also defined its role in BC, which is known for its extensive heterogeneity across morphological and molecular subtypes [9]. TB reflects an EMT‐like process associated with enhanced cell migration and apoptosis resistance, suggesting its association with tumor aggressiveness [12]. IHC analysis demonstrated that tumor bud cells showed upregulated vimentin expression and downregulated E‐cadherin expression relative to the tumor center, consistent with features of EMT [13].

Given the clinical importance of identifying reliable prognostic indicators, this study is aimed at exploring the potential relevance of TB in the context of IBC.

The study by Gujam et al. [8] reported the highest number of cases (*n* = 474).

A standardized method for assessing and reporting TB in colorectal adenocarcinoma was established by the International Tumor Budding Consensus Conference (ITBCC) and published in 2017, with endorsement from the College of American Pathologists (CAP) [11, 14]. In contrast, no such uniform guidelines currently exist for TB evaluation in breast carcinomas. In the present study including similar studies by Salhia et al. [15], Kumarguru et al. [9], Kundu et al. [16], and Muda et al. [17], *a* × 40 (high‐power) objective was used to identify and count tumor buds. In contrast, Liang et al. [18], and Gujam et al. [8] employed *a* × 20 objective. However, at ×20 magnification, distinguishing tumor buds from mimickers such as fibroblasts or inflammatory cells on H&E‐stained sections becomes challenging. To address this, Liang et al. utilized IHC for improved accuracy [18]. Therefore, evaluating TB under *a* × 40 objective on H&E sections is recommended to minimize misidentification and ensure accurate assessment [9, 18].

Present study along with other studies by Salhia et al. [15], Kumarguru et al. [9], Kundu et al. [16], and Muda et al. [17] assessed TB across 10 HPFs, whereas Liang et al. [18], Gujam et al. [8], used five HPF. With our experience, we recommend counting tumor buds in 10 fields because it offers greater accuracy and reproducibility.

As such, there is no standardized grading protocol for TB in breast carcinoma. Similar to the approaches used by Gujam et al. [8], Kumarguru et al. [9], and Muda et al. [17], we employed a cutoff of 20 tumor buds to classify cases into two groups: LTB (≤ 20 buds per 10 HPF) and HTB (> 20 buds per 10 HPF). In contrast, Francis et al. [19] classified TB as low (< 10) or high (> 10) per 20 HPF. The demographic characteristics, study design, and TB assessment parameters reported across major published studies are summarized in Table 4.

**Table 4:** Sociodemographic profile and tumor budding parameters in various studies in literature.

Parameters	Liang et al. [18]	Gujam et al. [8]	Salhia et al. [15]	Gabal et al. [20]	Agarwal et al. [21]	Masilamani et al. [22]	Kumarguru et al. [9]	Present study
Study origin	China	United Kingdom	Switzerland	Egypt	India	India	India	India
Study type	Retrospective	Retrospective	Retrospective	Cross‐sectional observational	Prospective	Retrospective	Retrospective	Retrospective
Year	2013	2015	2015	2018	2019	2019	2020	2025
*n*	160	474	148	61	40	107	50	229
Power of objective	20x	20x	40x	20x	20x	10x	40x	40x
Number of fields	5	5	10	10		10	10	10
Cutoff value for high tumor budding	> 7	> 20	> 4	> 4	> 10	> 10	> 20	> 20
Staining	H&E and IHC (AE1/AE3)	H&E	H&E and IHC (AE1/AE3)	H&E	H&E and IHC (AE1/AE3)	H &E	H&E	H&E and IHC AE1/AE3
Age (years)	12 ≤ 35/148 > 35	140 ≤ 50/334 > 50	Median 61 (32–91)	Mean ± SD 53.1 ± 12.6	24 ≤ 50/16 > 50	Median 53.7 (32–80)	Mean 53.14	Median: 50
Tumor size	< 5 cm:88.15	< 5 cm:97%	< 5 cm:95.3%	< 5 cm:70.5%	< 5 cm:67.5%	< 5 cm:50.5%	NA	< 2 cm: 8%
	> 5 cm:11.9%	> 5 cm:3%	> 5 cm: 4.7%	> 5 cm: 29.5%	> 5 cm:32.5%	> 5 cm:49.5%		2–5 cm: 76%
								> 5 cm: 16%
Tumor grade (most common)	Grade 2	Grades 2 and 3	Grade 2	Grade 1	Grade 3	Grade 2	NA	Grade 3

Renuka et al. followed the ITBCC 2016 guidelines. Tumor buds were assessed in a single hotspot (0.785 mm^2^) at the invasive front, with HTB defined as > 4 buds per 0.785 mm^2^ hotspot [23]. Liang et al. employed receiver operating characteristic (ROC) analysis and identified a cutoff of 7 tumor buds per 0.950 mm^2^ field, which was indicative of HTB [18].

Gujam et al. [8] evaluated tumor buds on H&E sections, whereas Salhia et al. [15], Sriwidyani et al. [24], and Liang et al. [18] used AE1/AE3 IHC, with the latter combining both methods. In this study, TB was mainly evaluated using H&E‐stained sections, whereas AE1/AE3 immunostaining was employed only in cases where bud counts were challenging to assess. Therefore, H&E staining remains appropriate for routine TB evaluation, whereas IHC should be reserved for cases with diagnostic uncertainty or borderline bud counts. Considering the clinical relevance of HTB, selective use of IHC is recommended.

This study demonstrated significant associations between TB and several adverse clinicopathological and molecular characteristics of BC, as elaborated further. Since age and tumor size are well‐established prognostic parameters in multiple malignancies, their relationship with TB was also examined. Both intratumoral budding (ITB) and peri‐tumoral budding (PTB) tended to be of a higher grade in older patients which was reported by Liang et al. [18] and Gujam et al. [8]. In contrast, present study, Kumarguru et al. [9], Shah et al. [25], and Sharma et al. [19] reported no significant correlation between TB and age of the patient. Continuous analysis further supported the lack of significant association between TB and patient age, tumor size, or pT stage, consistent with the categorical analysis and with findings reported by Kumarguru et al. [9], Shah et al. [25], and Sharma et al. [19]. In this study, most tumors measured 2–5 cm, aligning with the findings of Agarwal et al. [24], Francis et al. [17], and Singh et al. [25], who also reported a predominance of tumors ≤ 5 cm. In contrast, Gujam et al. [8] observed that most tumors were ≤ 2 cm, likely reflecting earlier detection in the United Kingdom due to better screening and awareness programs. A significant association between tumor size and TB has been reported by Liang et al. [18], Chandana et al. [26], and Agarwal et al. [21]. In contrast, Gabal et al. [20], Salhia et al. [15], Gujam et al. [8], and the present study did not demonstrate such a correlation, possibly due to differences in the methodologies used for tumor bud assessment. Consequently, the T stage, which is determined by tumor size in BC was also not significantly associated with TB in the present study, whereas studies by Kumarguru et al. [9], Liang et al. [18], Sriwidyani et al. [24], Muda et al. [17] and Agarwal et al. [21] reported a significant association. In the present study most of the cases were of Nottingham Grade 3, similar to study by Agarwal et al. [21], Muda et al. [17], and Rathod et al. [27], whereas Grade 2 was most common in studies by Liang et al. [18] and Sharma et al. [19]. A systemic review by Llyod et al. [10] including the studies by Singh et al. [28], Kumarguru et al. [9], Gujam et al. [8], Liang et al. [18], including the present study found no significant association between HTB and histologic grade; however, Sriwidyani et al. [24] and Shah et al. [25] reported a significant correlation. Shah et al. [25] reported that Grade 1 tumors exhibited LTB compared with Grade 3 tumors, which demonstrated HTB.

Although no significant association was found between tumor type and TB in the present study, all five ILC cases demonstrated HTB. E‐cadherin loss, a hallmark of EMT, is frequently observed in ILC and diffuse‐type gastric adenocarcinoma, both characterized by aggressive tissue infiltration. Moreover, ILC typically exhibits higher ER and PR expression with lower HER2 expression compared with stage‐matched ductal carcinomas, suggesting that HR positivity may contribute to enhanced EMT and promote TB.

In the present study, 132 cases (58%) of BC demonstrated associated DCIS, and the grade of this associated DCIS was evaluated for its correlation with TB. However, no significant association was identified. Continuous analysis similarly showed no significant correlation between DCIS grade and TB. The absence of a significant association between DCIS grade and TB suggests that TB may be more closely related to the invasive behavior of the carcinoma than to the grade of the associated in situ component. To date, no studies have specifically addressed this correlation, highlighting the need for further research in this area.

HTB in BC is associated with an increased incidence of LN metastasis and LVI. In the present study, LVI is identified in 35% of the cases, which was similar to the findings of Sharma et al. [19], Agarwal et al. [21], Liang et al. [18], and Salhia et al. [15]. Axillary LN status is a key prognostic factor in breast carcinoma. In the present study, 48% of the cases presented with LN metastasis. Similarly, in a study by Sharma et al. [19], LN metastasis was seen in 58.6% of cases [19]. HTB has been shown to be significantly associated with LN staging in studies by Salhia et al. [15], Kumarguru et al. [9], and Chandana et al. [26]. Similarly, in the present study, we observed a significant correlation between TB and N stage, suggesting that HTB is a risk factor for advanced nodal involvement. The observed association between TB burden and LN metastasis, LVI, and advanced nodal stage supports the concept that increasing TB reflects greater invasive and metastatic potential in breast carcinoma.

In present study, PNI was identified in 28 cases. PNI offers an alternative route of tumor spread beyond the conventional lymphatic and vascular pathways in BC. Through neurotrophic signaling and EMT, it contributes to tumor progression and locoregional recurrence (LRR). However, its independent prognostic significance in BC remains unclear due to insufficient large‐scale evidence [29]. In our study, we did not find any significant association between PNI and TB similar to Kumarguru et al. [9], whereas Shah et al. [25] observed a borderline significant correlation, with higher PNI rates in tumors with increased TB. Although the categorical comparison between PNI and TB did not reach statistical significance in our study, continuous analysis showed a weak but significant positive correlation between TB and PNI. This finding indicates that HTB counts may be associated with the presence of PNI, even though the association was not evident after dichotomization into low and HTB groups.

Necrosis is a recognized marker of aggressive BC biology, often observed in rapidly proliferating and hypoxic tumors. Although Kumarguru et al. [9], Chandana et al. [26] and Singh et al. [28] found a strong correlation between HTB and necrosis, this relationship was not significant in the present study or in the findings of Gujam et al. [8]. Similarly, continuous analysis showed no significant correlation between necrosis and TB.

Multiple studies have reported a higher prevalence of HTB in ER+/PR+ tumors, suggesting a potential hormone‐driven influence on EMT. In a systematic review by Llyod et al. [10], which included seven studies, ER and PR positivity were found to be significantly associated with the HTB group, whereas TNBC was associated with the LTB group. These findings align with the present study, which similarly demonstrated a significant correlation between ER and PR positivity with HTB, and TNBC with LTB. Rathod et al. [27] and Gujam et al. [8] also demonstrated a significant association between ER‐positive tumors and HTB. In contrast, Agarwal et al. [21] reported no correlation between ER, PR, or HER2/Neu expression and high tumor bud density. Masilamani et al. [22] identified a significant relationship between HER2/Neu expression and HTB, although they found no significant correlation with ER and PR expression. In the present study too, no significant correlation was found between HER2/Neu positivity and TB. Continuous analysis additionally demonstrated significant positive correlations between TB counts and ER positivity as well as PR positivity, whereas TNBC showed a significant negative correlation with TB. Molecular subtype analysis based on ER, PR, and HER2 status did not show a statistically significant overall association with TB, although the HR+/HER2− subtype was more frequent in the HTB group and TNBC was relatively more common in the LTB group. The predominance of HTB in HR positive BC implies that both TB and EMT may be regulated by hormonal influences. Several pathways, including Wnt, JAG2, TGF‐*β*, and growth factors, are known to drive EMT. ER*α* activation promotes EMT‐associated proteins and matrix metalloproteinases, thereby enhancing cell motility, suppressing E‐cadherin expression, and facilitating extracellular matrix degradation and tumor invasion [30].

HTB consists of small clusters of tumor cells that lose their normal adhesion and gain mobility, representing an early step in cancer spread. In a study of 47 BC cases, tumors with HTB showed elevated EMT markers (SNAIL, TWIST, N‐cadherin), whereas LTB tumors retained E‐cadherin expression, highlighting a strong link with invasive potential [31]. A meta‐analysis of nine datasets from eight studies demonstrated that HTB was significantly associated with poorer PFS [4]. Liang et al. [18] reported that patients with HTB had significantly poorer OS compared with those with LTB. Similarly, Sun et al. [32] found that HTB was associated with markedly reduced disease‐free survival (DFS) and OS. Gujam et al. [8] observed a shorter mean cancer‐specific survival (CSS) in patients with high‐grade TB, and noted that even among node‐negative patients, HTB cases remained linked to decreased CSS. Li et al. [33] demonstrated a significant association between HTB and the occurrence of distant metastases in ER‐positive/HER2‐negative BC, along with reduced DFS in this subgroup. Collectively, these studies suggest that HTB is associated with poorer OS, DFS, and CSS, along with an increased risk of distant metastasis. However, in the present study, TB did not emerge as an independent predictor of PFS or OS in the clinically adjusted multivariate Cox model. This suggests that the prognostic impact of TB may be mediated through its association with established adverse clinicopathological features, particularly LN metastasis, LVI, and advanced nodal stage. Consistent with this interpretation, pN stage remained the strongest independent prognostic factor, with pN3 disease being associated with a significantly higher risk of progression and death compared with pN0 disease. Nevertheless, HTB demonstrated significant associations with adverse clinicopathological parameters, including LN metastasis, LVI, advanced nodal stage, and HR positivity. The lack of association between TB and patient age, tumor size, and pT stage in the present study is consistent with the findings reported by Kumarguru et al. [9], Shah et al. [25], and Sharma et al. [19]. A comparison of the correlations between TB and clinicopathological parameters reported in the present and previous studies is summarized in Table 5.

**Table 5:** Correlation (*p* value) between tumor budding grade and various clinicopathological parameters in literature (*p* < 0.05: significant correlation, *p* < 0.001: highly significant correlation, *p* > 0.05: no significant correlation).

Parameters	Liang et al. [18]	Gujam et al. [8]	Salhia et al. [15]	Agarwal et al. [21]	Masilamani et al. [22]	Kumarguru et al. [9]	Present study
Age	0.513	0.080	NA	NA	NA	0.729	0.406
Tumor size	**0.014**	0.469	NA	**0.03**	NA	NA	0.894
Tumor grade	0.163	0.099	**0.036**	0.233	**> 0.05**	0.884	0.107
Lymphovascular invasion (LVI)	**< 0.001**	**< 0.001**	**0.015**	**0.02**	**< 0.05**	**< 0.001**	**< 0.001**
Perineural invasion (PNI)	NA	0.107	0.7	NA	NA	0.589	**0.073**
Necrosis	NA	0.078	NA	0.283	NA	**0.004**	0.359
Lymph node metastasis/pN stage	**0.05**	**0.009**	**0.03**	0.601	**< 0.05**	**< 0.001**	**< 0.05**
Estrogen receptor (ER)	0.28	**0.003**	**0.02**	0.505	**> 0.05**	NA	**0.027**
Progesterone receptor (PR)	0.28	0.054	NA	0.787	**> 0.05**	NA	**0.018**
HER2/Neu	0.1	0.429	0.68	0.991	**< 0.05**	NA	0.428

*Note:* Bold *p* values are statistically significant.

## 6. Limitations of the Study

The present study has certain limitations. Being a retrospective single‐center study, the findings may have limited generalizability. Formal interobserver reproducibility analysis for TB assessment was not performed, although discrepant cases were reviewed jointly to achieve consensus. TB was categorized using a cutoff derived from previously published literature to facilitate interstudy comparability; however, cohort‐specific ROC‐based optimization was not performed. Additionally, no significant association between TB and PFS or OS was observed in the present cohort.

## 7. Conclusion

TB is a cost‐effective histopathological marker that shows significant associations with adverse clinicopathological features, including LN metastasis, LVI, advanced nodal stage, and distant metastasis in BC. Although no significant association with PFS or OS was demonstrated in the present cohort, the observed correlations with established adverse pathological parameters suggest that TB may serve as a useful indicator of aggressive tumor behavior. Further prospective studies with standardized assessment protocols are warranted to better define its clinical utility.

## Funding

No funding was received for this manuscript.

## Conflicts of Interest

The authors declare no conflicts of interest.

## Data Availability

The data that support the findings of this study are available on request from the corresponding author. The data are not publicly available due to privacy or ethical restrictions.
